# 2084: Knowledge, attitudes, and experiences towards genetic research among persons of African descent

**DOI:** 10.1017/cts.2017.189

**Published:** 2018-05-10

**Authors:** Jane Otado, Veronica Thomas, Shawneequa Callier, Faun Rockcliffe, Dietrich Johnson, Denise Scott

## Abstract

OBJECTIVES/SPECIFIC AIMS: The purpose of this descriptive study is to explore knowledge, attitudes, and behaviors related to genetics and genetic research in a sample of persons of African descent. METHODS/STUDY POPULATION: Data were generated using a cross-sectional survey design. A nonprobability sample of 272 persons of African descent, ages 18 and older, were recruited from the Washington, DC metropolitan area through public advertisement and word-of-mouth. Participants had diverse backgrounds with most born in the United States (93%), female (71%), some college or above education (57%), household income under $40,000 (54%), and some with a reported disability (38%). Before survey recruitment and administration, this study was reviewed and approved by the Howard University Institutional Review Board. RESULTS/ANTICIPATED RESULTS: The majority (79.8%) of the participants considered themselves as having a “fair” to “good” knowledge of genetics. The sample had a 2.24 (SD=77) mean score on the 5-item genetics knowledge questionnaire with total possible mean scores ranging from 0 (no correct responses) to 5 (all correct responses). Most (53.3%) participants believe it is important for persons of African descent to participate in genetic research. However, almost one-half (46.7%) felt that information from genetic research can be used to discriminate against minorities. In terms of behaviors, 83.4% of the participants never had genetic testing conducted. However, an overwhelming majority reported that they would be willing to participate in a genetic research project specifically for detection of risk factors such as cancer (87%), diabetes (89.3%), Alzheimer disease (88.6%), and alcohol use disorder (75%). DISCUSSION/SIGNIFICANCE OF IMPACT: This investigation suggests that persons of African descent generally view participation in genetic research as important and are willing to have their genetic profile analyzed to detect susceptibility to certain diseases. However, ethical issues, such as misuse of genetic research to discriminate against minorities, remain a prominent concern. Further studies are needed to illuminate KABEs and to help identify the role these factors may play in this population’s willingness to participate in testing and research. Such information could provide invaluable insight to the development and implementation of more ethical and culturally competence strategies for recruiting minority participants into genetic research.

